# State-Dependent FGF Signaling in Satellite Cell-Mediated Skeletal Muscle Regeneration and Pathological Remodeling

**DOI:** 10.3390/biom16091238

**Published:** 2026-08-26

**Authors:** Shuying Fu, Yuhuan Meng, Keying Liang, Meiying Feng

**Affiliations:** 1 School of Life Sciences, Zhaoqing University, Zhaoqing 526061, China; fushuying@zqu.edu.cn; 2 Guangzhou KingMed Transformative Medicine Institute & KingMed School of Laboratory Medicine, Guangzhou Medical University, Guangzhou 511436, China; mengyuhuan@gzhmu.edu.cn; 3 Guangzhou Municipal and Guangdong Provincial Key Laboratory of Protein Modification and Degradation, School of Basic Medical Sciences, Guangzhou Medical University, Guangzhou 511436, China; keyingliang@gzhmu.edu.cn

**Keywords:** fibroblast growth factor, state-dependent signaling, muscle stem cells, skeletal muscle regeneration, regenerative niche, aging, pathological remodeling, rhabdomyosarcoma

## Abstract

Skeletal muscle regeneration depends on coordinated transitions of muscle stem cells (MuSCs), also known as satellite cells, from quiescence through activation and proliferative expansion to differentiation and fusion, while self-renewal replenishes the quiescent MuSC pool within a dynamically remodeled niche. Fibroblast growth factor (FGF) signaling regulates these transitions, but its effects vary as MuSCs and their niche change across regenerative stages. FGF output is shaped by ligand availability and extracellular presentation, fibroblast growth factor receptor (FGFR) isoform expression and coreceptor availability, receptor trafficking, intracellular feedback, and the state of the responding cell. Following acute injury, FGF inputs can support MuSC activation and expansion; signaling is subsequently reconfigured during differentiation, fusion, self-renewal, and return to quiescence. Aging-associated regenerative decline, chronic injury and dystrophic remodeling, denervation, and metabolic dysfunction disrupt this coordination and can uncouple FGF activity from productive repair. Rhabdomyosarcoma provides a distinct malignant context in which the FGF network is rewired to sustain oncogenic myogenic cell states. Here, we integrate molecular, cellular, and niche-level evidence across these settings to explain why FGF signaling produces divergent outcomes and to clarify how cellular context and timing should inform therapeutic modulation.

## 1. Introduction

Skeletal muscle constitutes approximately 40% of adult body mass and plays essential roles in locomotion, posture, whole-body metabolism, and thermoregulation [1,2,3]. Beyond its contractile and metabolic functions, skeletal muscle has a remarkable capacity to restore tissue structure and function after injury [4]. This regenerative capacity depends largely on muscle stem cells (MuSCs), also known as satellite cells, which reside between the sarcolemma of myofibers and the basal lamina [5]. Under homeostatic conditions, most MuSCs remain quiescent. In response to injury, they enter the cell cycle and generate myogenic progeny that either differentiate and fuse to repair damaged myofibers or self-renew to replenish the stem cell pool [6,7]. Efficient regeneration therefore requires the coordinated progression of MuSC activation, proliferative expansion, differentiation, fusion, and return to quiescence [8,9].

MuSC fate transitions are not governed solely by cell-intrinsic programs but are continuously shaped by local niche cues and systemic physiological states [8,9,10]. Among the signaling pathways involved in skeletal muscle regeneration, fibroblast growth factor (FGF) signaling has long been recognized as an important regulator of MuSC behavior [11,12]. FGF signaling contributes to multiple stages of MuSC-mediated repair, but its biological effects are not determined by ligand abundance alone. Rather, FGF responsiveness changes as MuSC state and niche conditions evolve across successive stages of regeneration [12,13]. Variation in extracellular ligand presentation and receptor competence contributes to this state dependence [14,15,16]. More broadly, FGF output depends on the properties of the ligand input, including its source, concentration, timing, and duration, as well as on ligand specificity for fibroblast growth factor receptor (FGFR) isoforms and coreceptor availability [17,18]. Receptor trafficking and complex assembly, intracellular feedback regulation, and the signaling state of the responding cell further shape signal amplitude, duration, and biological outcome [19,20]. Here, state-dependent FGF signaling refers to the integration of ligand input, extracellular presentation, receptor competence, and intracellular signal regulation with the changing state of MuSCs and their niche across successive stages of regeneration.

Previous reviews have established that MuSC fate and function are regulated by dynamic interactions with the niche [8,9,21,22]. In skeletal muscle, an FGF-focused review has discussed the role of FGF signaling in MuSC behavior and regeneration, together with cell-surface and extracellular mechanisms that modulate FGF responsiveness, particularly during aging [12]. Broader reviews of the FGF system have further shown that signaling output is shaped by ligand–receptor specificity, interactions with cofactors and coreceptors, and cellular context [17,18,19,20]. Building on this foundation, the present review integrates these molecular and niche-level determinants within a state-dependent framework centered on successive MuSC fate transitions. We extend this framework to aging-associated decline and pathological muscle remodeling, examining how changes in niche conditions and cellular state alter FGF signal presentation, reception, and interpretation. We distinguish evidence derived from myogenic cell lines, primary MuSC cultures, animal models, and human studies, while also considering whether findings directly involve MuSCs or reflect changes at the myofiber, niche, or systemic level.

In this review, we first define the molecular determinants that govern FGF ligand availability and signal reception in regenerating muscle. We then examine how FGF signaling is interpreted across successive MuSC fate transitions during regeneration. Finally, we discuss how FGF signaling is altered during aging-associated decline and in conditions associated with pathological muscle remodeling, including chronic injury and dystrophic remodeling, denervation, and metabolic dysfunction. We also consider rhabdomyosarcoma (RMS) separately as a malignant myogenic context characterized by aberrant FGF signaling.

Relevant literature was identified by searching PubMed, Web of Science, and Scopus from database inception through August 2026. Search terms included fibroblast growth factor, FGF, FGFR, muscle stem cell, satellite cell, myoblast, and skeletal muscle regeneration, together with terms related to MuSC fate transitions, including quiescence, activation, proliferation, differentiation, fusion, and self-renewal. Additional terms reflected the conditions discussed in this review, including aging, chronic injury, muscular dystrophy, denervation, diabetes, metabolic dysfunction, and rhabdomyosarcoma. Reference lists of relevant articles were also screened. Publications were selected if they provided relevant evidence on FGF signaling in MuSCs or myogenic models, its role in skeletal muscle regeneration, or alterations in FGF signaling under the conditions covered in this review. Mechanistic studies outside skeletal muscle were also included when they informed FGF–FGFR interactions, cofactor and coreceptor involvement, receptor trafficking and complex assembly, or intracellular signal regulation.

## 2. Molecular Determinants of State-Dependent FGF Signaling in Skeletal Muscle Regeneration

FGFs regulate development, tissue homeostasis, repair, regeneration, and metabolic adaptation [19,20,23,24]. During skeletal muscle regeneration, however, ligand expression alone does not predict signaling output. FGF responsiveness depends on ligand source, concentration, duration, and spatial range; the availability of compatible FGFRs, cofactors, and coreceptors; and the way in which extracellular presentation, receptor trafficking, and intracellular feedback shape signal amplitude and persistence [12,17,18]. These variables change as injury progresses and MuSCs move between quiescence, activation, expansion, and lineage progression. The molecular basis of state dependence can therefore be considered at three interconnected levels: ligand diversity and spatial range, receptor and coreceptor composition, and niche-dependent control of ligand availability and presentation.

### 2.1. Ligand Diversity Defines the Source and Spatial Range of FGF Signaling

The mammalian FGF family comprises 22 proteins organized into seven phylogenetic subfamilies [25,26]. Human FGFs are designated FGF1–FGF14 and FGF16–FGF23; mouse FGF15 is orthologous to human FGF19 and is commonly designated FGF15/FGF19 in cross-species discussions [27,28]. Table 1 summarizes the subfamily relationships, receptor preferences, and principal cofactor requirements relevant to interpreting FGF signaling [18,20,29,30].

Intracellular FGFs correspond to the FGF11 subfamily, also known as FGF homologous factors (FHFs) [30]. They lack conventional N-terminal secretion signals and primarily function intracellularly through protein–protein interactions rather than as classical FGFR ligands [30,31,32]. Recent studies in non-muscle cell systems have nevertheless shown that FGF11–FGF14 can undergo unconventional extracellular release and, once extracellular, bind FGFR1 and activate downstream signaling, including anti-apoptotic responses [33,34,35]. Whether this mode of signaling occurs in regenerating skeletal muscle remains unknown. Muscle-related evidence is currently limited mainly to the C2C12 myoblast cell line, in which FGF13 overexpression inhibited proliferation and differentiation and was associated with reduced expression of the receptor tyrosine kinase (RTK) signaling antagonist SPRY1 and altered extracellular signal-regulated kinase 1/2 (ERK1/2) activity [36]. Direct evidence linking FHFs to adult MuSC-mediated regeneration remains limited, so the following discussion focuses primarily on secreted FGFs. Paracrine FGFs comprise the FGF1, FGF4, FGF7, FGF8, and FGF9 subfamilies [37,38]. These ligands act extracellularly, although FGF1 and FGF2 lack conventional signal peptides and reach the extracellular space through nonclassical release mechanisms [37,38].

Paracrine FGFs contain heparin-binding domains that confer moderate to high affinity for heparan sulfate (HS) chains carried by heparan sulfate proteoglycans (HSPGs), including transmembrane syndecans, glypicans, and extracellular matrix (ECM)-associated proteoglycans such as perlecan, agrin, and collagen XVIII [19,39,40,41]. For MuSCs, the biologically relevant input is therefore determined not only by which FGF is produced but also by its local concentration, persistence, and accessibility within the regenerative niche [18,41,42,43]. Experiments in non-muscle cells further show that FGF2 concentration and HS availability can independently alter FGFR substrate 2α (FRS2α) phosphorylation and ERK signaling kinetics [44]. Comparable quantitative relationships between ligand dose, exposure duration, and signaling outcome have not yet been systematically defined in adult MuSCs.

Endocrine FGFs of the FGF15/19 subfamily follow a different signaling logic. Their reduced affinity for HS limits local ECM retention and facilitates endocrine distribution, whereas weak intrinsic FGFR binding creates a requirement for Klotho coreceptors [45,46]. FGF15/19 and FGF21 signal primarily with β-Klotho, whereas FGF23 depends mainly on α-Klotho [47,48]. Endocrine FGFs can therefore connect systemic metabolic or hormonal states to skeletal muscle. Circulating ligand abundance, however, does not by itself establish a direct effect on MuSCs; such an inference additionally requires evidence that the relevant cell population expresses a signaling-competent FGFR–Klotho complex.

### 2.2. FGFR Isoforms and Coreceptor Context Shape FGF Signal Reception

**Figure 1:**
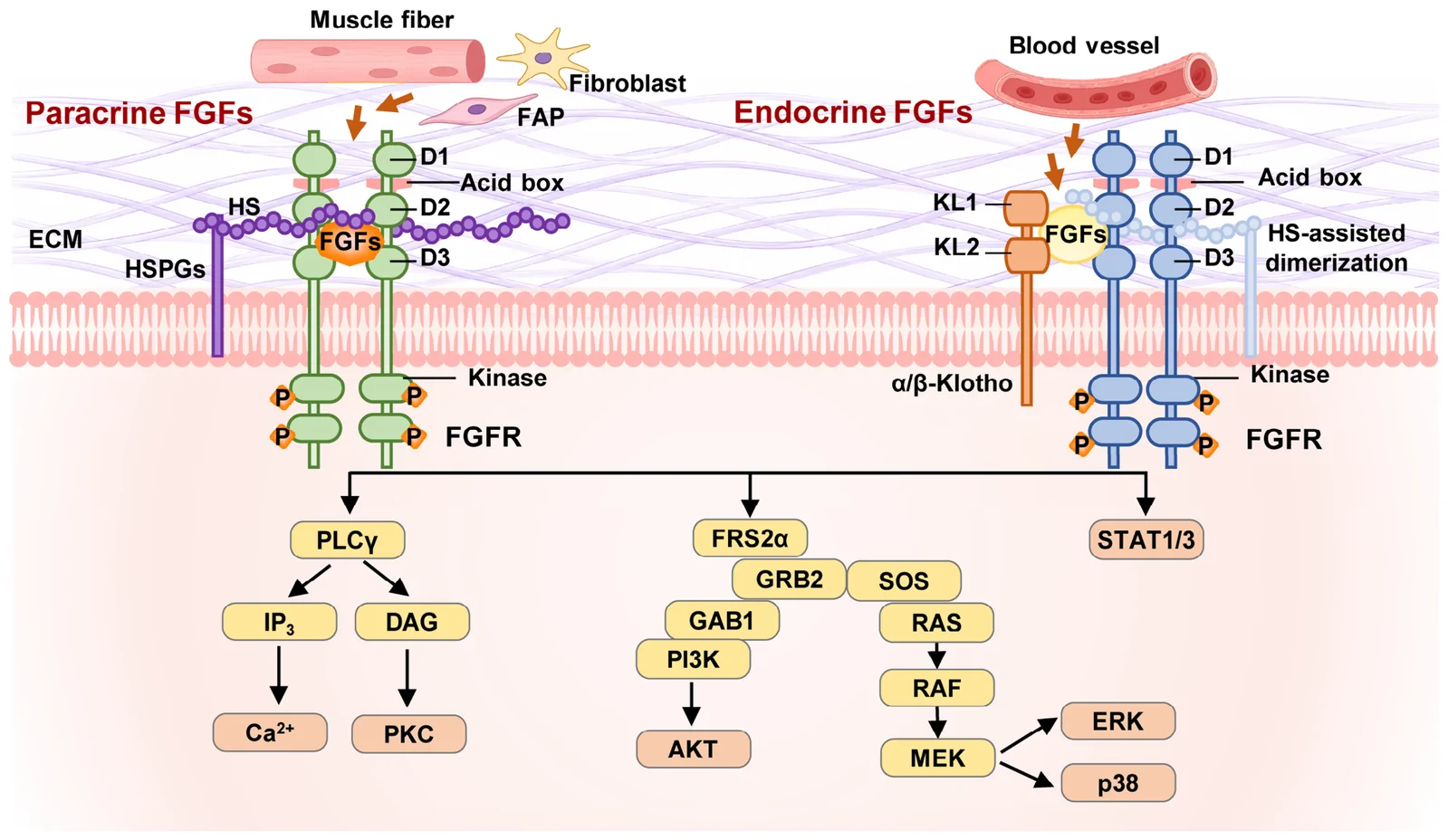
Molecular organization of FGF signal reception and downstream signaling in skeletal muscle regeneration. Paracrine FGFs are locally retained and presented by extracellular matrix (ECM)-associated HS/HSPGs, which also support productive FGF–FGFR complex assembly. Endocrine FGFs have lower HS affinity and signal through compatible FGFR–α-Klotho or FGFR–β-Klotho complexes, with HS contributing to receptor-complex stabilization and dimerization in defined systems. FGFRs contain three extracellular immunoglobulin-like domains (D1–D3), an acid box in the D1–D2 linker, a transmembrane segment, and an intracellular kinase domain; Klotho coreceptors are shown with their extracellular Klotho-like 1 and 2 (KL1 and KL2) domains. Ligand binding promotes FGFR dimerization and phosphorylation, leading to activation of major downstream pathways, including phospholipase C gamma (PLCγ)–inositol 1,4,5-trisphosphate (IP_3_)/diacylglycerol (DAG)–Ca^2+^/protein kinase C (PKC), FGFR substrate 2α (FRS2α)–growth factor receptor-bound protein 2 (GRB2)/son of sevenless (SOS)–RAS–RAF–MEK/mitogen-activated protein kinase (MAPK) signaling with extracellular signal-regulated kinase (ERK) and p38 MAPK outputs, GRB2-associated binding protein 1 (GAB1)–phosphoinositide 3-kinase (PI3K)–protein kinase B (AKT), and signal transducer and activator of transcription 1/3 (STAT1/3) signaling. These signaling modules provide the molecular basis for state-dependent FGF interpretation during skeletal muscle regeneration.

Ligand availability produces a cellular response only when the responding cell can assemble a compatible receptor complex [19,49]. FGFR1–FGFR4 are RTKs with three extracellular immunoglobulin-like domains (D1–D3), a single transmembrane helix, and an intracellular kinase domain (Figure 1) [18,50]. D2 and D3 form the principal ligand-binding region, whereas the D1–D2 linker contains an acidic segment, termed the acid box, that contributes to receptor autoinhibition [51,52]. Alternative splicing within D3 generates IIIb and IIIc variants of FGFR1–FGFR3, whereas FGFR4 lacks this splice distinction [53,54]. These receptor isoforms display distinct but overlapping ligand preferences, and their cell-type- and state-dependent expression determines which components of the extracellular FGF repertoire can be received [19,29,49]. FGFR1 and FGFR4 provide the clearest direct examples of receptor competence in adult MuSCs. Genetic studies indicate partially overlapping functions of these receptors: myogenic-lineage loss of FGFR1 markedly reduces FGF2 responsiveness, whereas combined MuSC-specific loss of FGFR1 and FGFR4 produces broader defects in MuSC homeostasis and regeneration [13,55]. These findings illustrate how receptor composition can shape the interpretation of an FGF-rich niche without treating receptor expression and ligand abundance as interchangeable evidence.

For paracrine FGFs, HS stabilizes productive FGF–FGFR interactions and facilitates receptor dimerization [40,56,57]. Structural studies have proposed different symmetric and asymmetric arrangements of FGF–FGFR–HS complexes, but collectively support a direct role for HS in productive receptor complex assembly [58,59]. Endocrine FGFs additionally require α- or β-Klotho to stabilize ligand–FGFR recognition, with HS also contributing to receptor assembly and activation [45,46,60]. This cooperative role of Klotho and HS is structurally best defined in the FGF23 system, in which HS promotes assembly and dimerization of the FGF23–FGFR–α-Klotho complex [61,62,63]. Productive receptor activation therefore depends on the compatibility of ligand, FGFR, coreceptor, and extracellular cofactor context rather than on a simple ligand–receptor interaction [64,65].

Receptor assembly brings the intracellular kinase domains into proximity, leading to ordered transphosphorylation, relief of kinase autoinhibition, and formation of docking sites for signaling proteins [66,67,68]. Two major proximal effectors are FRS2α and phospholipase Cγ (PLCγ) [69,70]. Phosphorylated FRS2α recruits growth factor receptor-bound protein 2 (GRB2), linking FGFR activation through son of sevenless (SOS) to RAS–RAF–MEK/MAPK signaling, including ERK and context-dependent p38 MAPK outputs [70,71,72]. FRS2α also links FGFRs to phosphoinositide 3-kinase (PI3K)–protein kinase B (AKT) signaling through GRB2-associated binding protein 1 (GAB1) [70,71]. In parallel, activated FGFRs can recruit and phosphorylate PLCγ, leading to inositol-1,4,5-trisphosphate (IP_3_)-dependent Ca^2+^ mobilization and diacylglycerol (DAG)-dependent protein kinase C (PKC) activation [69,73,74]. FGFR activation may also engage signal transducer and activator of transcription 1 and 3 (STAT1/3) in a ligand-, receptor-, and cell-state-dependent manner [20,75]. Among these downstream modules, ERK/MAPK signaling has the most consistent direct support as a functional FGFR effector in adult MuSC-related systems, whereas altered FGFR1–p38α/β MAPK signaling has been associated with impaired self-renewal in aged MuSCs [13,14,55,72]. Thus, FGF output is shaped not only by pathway identity but also by signal amplitude, duration, localization, and the signaling competence of the responding cell.

Receptor trafficking and feedback regulation further determine how long an extracellular input remains biologically active. Following activation, FGFRs undergo endocytosis and endosomal sorting; receptors may continue signaling from intracellular compartments, recycle to the cell surface, or be directed toward attenuation and degradation [76]. Sprouty proteins, similar expression to FGF (SEF)/interleukin 17 receptor D (IL17RD), dual-specificity MAPK phosphatases, and casitas B-lineage lymphoma (CBL)-mediated ubiquitination constrain receptor and ERK pathway activity at different levels [77,78,79,80]. In MuSCs, SPRY1 provides a direct muscle-relevant example of this feedback control [15,81]. By contrast, although FGFR trafficking is well characterized in other cellular systems, endosomal residence time and compartment-specific FGFR output have not been directly resolved in adult MuSCs. Exposure duration therefore reflects both the persistence of extracellular ligand and the capacity of the responding cell to sustain, recycle, or terminate receptor signaling.

### 2.3. The Regenerative Niche Shapes FGF Signal Availability and Presentation

The regenerative niche adds spatial and temporal regulation between ligand production and receptor activation. This layer is particularly important for paracrine FGFs, whose accessibility depends on HS-containing proteoglycans, ECM organization, and adhesion-associated cell-surface complexes [56,61,82]. Because these niche components are remodeled after injury, similar amounts of ligand can generate different effective exposures at different regenerative stages. Endocrine FGF input is less dependent on local ligand retention, but it remains contingent on the FGFR–Klotho competence of the responding cell.

HS chains on HSPGs act as extracellular reservoirs that regulate the retention, release, diffusion, and receptor engagement of paracrine FGFs [57,83,84]. During skeletal muscle differentiation and regeneration, HSPG abundance and distribution are dynamically remodeled [83,84,85]. Syndecan-3 and syndecan-4, for example, mark MuSCs and have non-redundant functions in MuSC maintenance and muscle repair [86,87]. HS is not a uniform polymer: the position and extent of sulfation determine the affinity and selectivity of FGF–FGFR assembly rather than simply increasing ligand binding [88,89,90]. The extracellular 6-O-endosulfatases Sulf1 and Sulf2 illustrate the functional importance of this regulation in muscle. By remodeling HS sulfation, Sulfs alter FGF availability and receptor engagement [91,92]. Changes in HS fine structure can therefore alter FGF localization, intensity, and duration without a corresponding change in ligand abundance.

FGF responsiveness is also integrated with adhesion and cell-surface organization. In mouse MuSCs, β1-integrin cooperates with FGF2 to activate ERK and AKT; impaired β1-integrin activity reduces FGF responsiveness [93]. MuSC-specific loss of Cdon likewise impairs integrin activation, FGFR signaling, and muscle regeneration, providing direct in vivo evidence that adhesion competence modifies FGF reception [14]. Fibronectin represents another important component of this adhesion-dependent niche and supports integrin-dependent organization and regenerative competence of MuSCs [94]. These findings connect FGFR activity to the physical organization of the MuSC surface rather than treating it as an isolated ligand–receptor event.

HSPGs are not functionally interchangeable, because their effects reflect both HS-dependent ligand binding and the membrane or ECM localization imposed by the core protein. Individual HSPGs can therefore facilitate or restrict FGF signaling by altering ligand retention, membrane partitioning, and receptor accessibility. In C2C12 myoblasts, glypican-1 partitions FGF2 into lipid raft microdomains and thereby limits its access to FGFRs [95]. In non-muscle cell systems, syndecan-4 can couple HS-dependent FGF signaling to cell-surface and adhesion-associated organization, whereas genetic studies of syndecan-3 and syndecan-4 demonstrate broader, distinct roles in MuSC homeostasis and regeneration [86,87,96,97,98]. Studies in avian satellite-cell cultures further show that changes in syndecan-4 and glypican-1 expression accompany altered FGF2 responsiveness, emphasizing that these relationships can vary with species, age, and experimental model [99]. Thus, the effect of an HSPG depends not only on its HS chains but also on core protein identity, membrane localization, and the state of the responding cell.

Taken together, these findings identify the regenerative niche as an active determinant of FGF signal reception rather than simply a source of ligands. Direct evidence in adult MuSCs supports the importance of receptor competence, adhesion-dependent modulation of FGF responsiveness, and SPRY1-mediated feedback restraint [13,14,15,55,81,93]. By contrast, quantitative relationships between ligand dose or exposure duration and signal output, as well as the contributions of HS-dependent presentation, receptor trafficking, and compartment-specific signaling, have been characterized mainly in non-muscle systems or myogenic cell models [44,76,88,91,95]. FGF responsiveness therefore emerges from the interplay among ligand input, receptor competence, niche presentation, and intracellular regulation, providing the molecular basis for understanding how FGF signaling is interpreted across successive MuSC fate transitions.

## 3. State-Dependent Regulation of MuSC Fate Transitions by FGF Signaling

**Figure 2:**
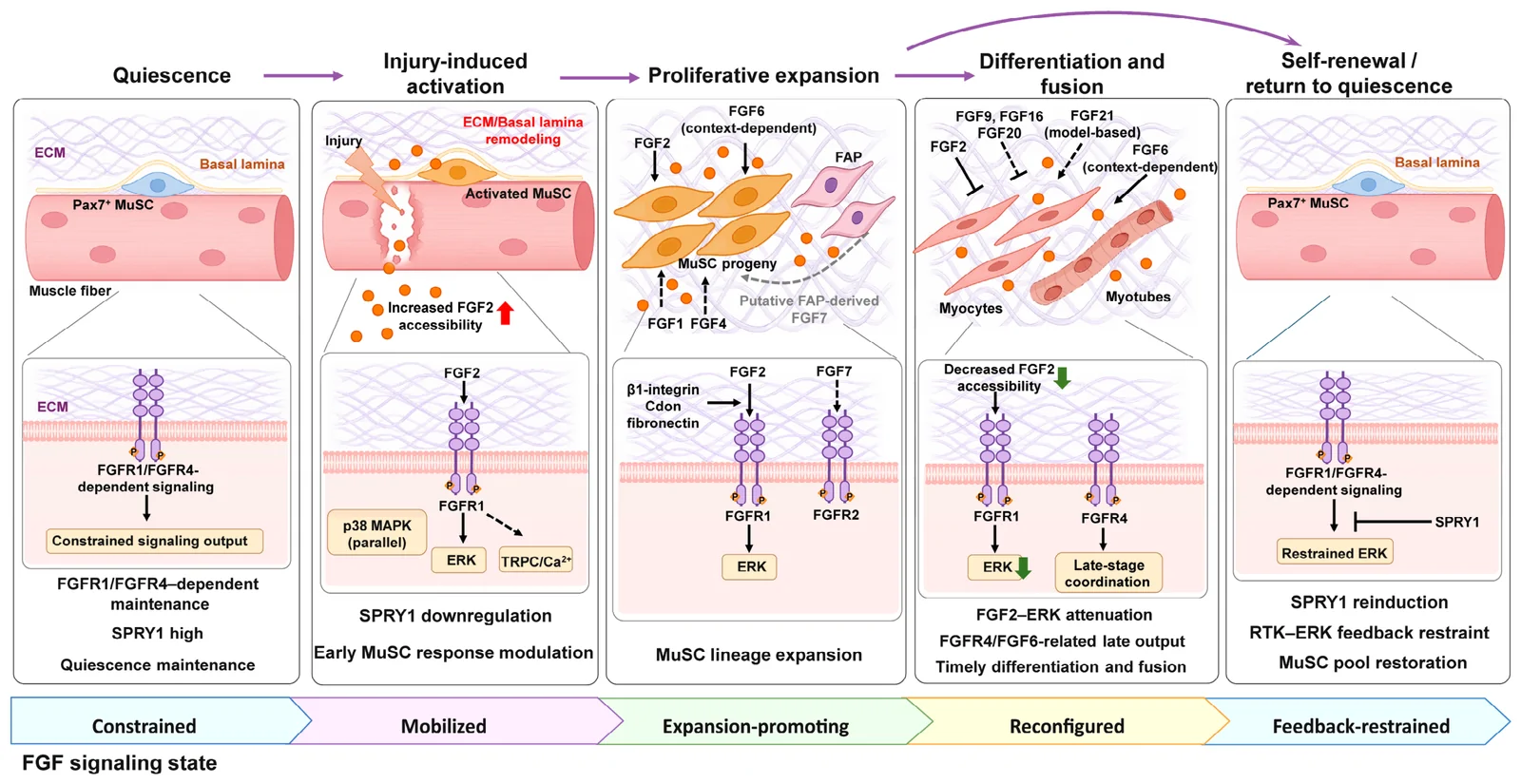
State-dependent FGF signaling during MuSC-mediated skeletal muscle regeneration. FGF signaling is interpreted differently across successive muscle stem cell (MuSC) fate transitions. During quiescence, constrained combined FGFR1/FGFR4-dependent signaling and high SPRY1 expression help maintain Pax7^+^ MuSCs and preserve the MuSC pool. After injury, myofiber damage and ECM/basal lamina remodeling increase FGF2 accessibility from damaged tissue and matrix-associated reservoirs, supporting FGF2–FGFR1-dependent early MuSC responses. During proliferative expansion, FGF2–FGFR1–ERK signaling and adhesion-associated niche regulators, including β1-integrin, Cdon, and fibronectin, support activated MuSC lineage expansion. The expansion-promoting effects of FGF1 and FGF4 are supported mainly by model-dependent evidence, whereas the putative fibro-adipogenic progenitor (FAP)-derived FGF7–FGFR2 axis remains an emerging niche interaction. During differentiation and fusion, reduced receptor-accessible FGF2 and attenuation or reconfiguration of FGFR1–ERK signaling accompany lineage progression. Model-based effects of FGF9, FGF16, FGF20, and FGF21, together with context-dependent FGF6 activity and FGFR4-dependent signaling, illustrate late-stage reconfiguration. SPRY1 reinduction restrains RTK-ERK signaling and supports restoration of the quiescent MuSC pool. Solid black connectors indicate relationships supported by direct functional evidence, including in vivo validation or convergent genetic and/or intervention studies; black dashed connectors indicate functional evidence derived mainly from primary-cell, ex vivo, or model-limited studies without comparable in vivo validation; and gray dashed connectors indicate associative, inferred, or emerging relationships without direct functional validation. Arrowheads denote promoting or contributing relationships, whereas blunt-ended bars denote inhibitory or restraining relationships.

Skeletal muscle regeneration proceeds through coordinated but overlapping MuSC fate transitions [8,9,100,101]. Activated MuSCs and their progeny display substantial heterogeneity in proliferative history, lineage commitment, and self-renewal potential [102,103,104,105]. Within this heterogeneous regenerative landscape, FGF signaling is interpreted differently across MuSC states. Figure 2 summarizes the principal FGF relationships across this regenerative sequence.

### 3.1. FGF Signaling in Quiescence Maintenance and Injury-Induced Activation

MuSC quiescence is an actively maintained and reversible state that preserves stem cell identity and long-term regenerative capacity [106,107]. Anchorage to the myofiber and basal lamina establishes the structural context in which quiescent MuSCs receive growth factor signals [108,109,110]. Within this context, maintenance of quiescence appears to require FGFR signaling that is present but appropriately constrained, rather than either complete pathway inactivity or persistent activation [13,55,111].

FGFR1 and FGFR4 are the predominant FGFRs detected in quiescent MuSCs and their activated progeny [13,55,112,113]. Earlier studies targeting either receptor alone produced comparatively mild phenotypes or relied on models that did not isolate a cell-autonomous requirement in adult MuSCs [13,55,111]. Myogenic-lineage deletion of *Fgfr1* markedly reduced FGF2-dependent proliferation of myofiber-associated MuSCs but did not abolish regeneration after acute cardiotoxin injury, whereas germline *Fgfr4* deficiency resulted in delayed and disorganized regeneration without resolving the MuSC-specific contribution [55,111]. By contrast, inducible MuSC-specific deletion of both *Fgfr1* and *Fgfr4* resulted in spontaneous loss of quiescence, precocious differentiation, and progressive depletion of the MuSC pool [13]. These findings establish an important role for combined FGFR1- and FGFR4-dependent signaling in preserving the uncommitted MuSC state, although the endogenous ligand inputs responsible for this receptor-dependent quiescent state remain unresolved.

Injury changes both the physical organization and signaling composition of the MuSC niche. Damage to myofibers, immune-cell recruitment, stromal-cell activation, and remodeling of the basal lamina and ECM alter the accessibility and persistence of locally retained growth factors [114,115,116]. For paracrine FGFs, changes in HSPG distribution, HS sulfation, and matrix architecture can modify ligand availability and presentation at the MuSC surface without requiring a corresponding change in total ligand expression [12,56,83,86,93]. Because quiescence exit integrates multiple injury-responsive cues, FGF signaling should be viewed as an important component of the early activation program rather than its sole initiating trigger [117,118,119,120].

FGF2 is among the best-characterized FGF inputs during the early regenerative response [55,121]. In isolated myofiber systems, exogenous FGF2 promotes recruitment of quiescent MuSCs into the regenerative response and induces transient receptor potential canonical (TRPC)-mediated Ca^2+^ influx and nuclear factor of activated T cells (NFAT) signaling in associated MuSCs [122,123]. In parallel, p38α/β MAPK regulates quiescence exit, myoblast determination protein 1 (MyoD) induction, and asymmetric fate allocation [124,125]. FGF2-associated ERK and Ca^2+^ signaling therefore operate alongside p38 MAPK-dependent regulation during early MuSC activation. *Spry1* expression also decreases as MuSCs enter the regenerative response, consistent with reduced feedback restraint on RTK signaling [15,81]. Early activation thus coincides with increased access to growth factor inputs and reduced intracellular feedback restraint.

### 3.2. FGF Signaling During MuSC Proliferative Expansion

Following activation, MuSCs undergo proliferative expansion to generate sufficient myogenic progenitors for tissue reconstruction while retaining a population capable of self-renewal [8,9,101]. This stage represents the most consistently documented mitogenic context for canonical FGF signaling in the MuSC lineage. Primary MuSC and myogenic culture studies have shown that several FGFs, including FGF1, FGF2, FGF4, and FGF6, can promote proliferation [112,126,127,128,129,130].

The strength and directness of the evidence, however, differ among ligands. FGF1 and FGF4 promote proliferation in cultured adult MuSCs, but direct evidence for their roles in adult muscle regeneration in vivo remains limited [127,129]. Among these ligands, FGF2 has the most extensive functional support for promoting expansion of adult MuSCs, whereas FGF6 has a distinct role in scaling the MuSC pool during early postnatal development [55,123,126,131]. FGF7 has more recently emerged as a candidate proliferative niche signal supported by primary cell studies and in vivo intervention [132].

In isolated myofiber and primary cell systems, FGF2 increases the proliferation of MuSCs [55,112,130]. The reduced response to FGF2 after myogenic-lineage deletion of *Fgfr1* supports FGFR1 as a major mitogenic receptor in adult MuSCs [55]. Consistent with this, MuSC-specific combined deletion of *Fgfr1* and *Fgfr4* impairs lineage expansion during regeneration and is associated with reduced ERK signaling [13,55]. The mitogenic FGF2–FGFR1 response is also conditioned by the adhesive state of the MuSC, including β1-integrin- and Cdon-dependent signaling competence and fibronectin-supported cell-surface organization [14,93,94]. FGF2 therefore functions as a niche-coupled expansion signal rather than simply as a soluble mitogen.

FGF6 also influences myogenic progenitor abundance, although its role in acute regeneration has varied among studies. One *Fgf6*-deficient mouse model displayed impaired regeneration and reduced numbers of activated myogenic cells, whereas another reported little overt impairment after injury [126,133]. Differences in genetic background and injury paradigm may contribute to these divergent findings [133,134]. More recent work identifies a distinct role for FGF6 in establishing the size of the adult MuSC pool during a critical postnatal window. *Fgf6* deficiency reduced adult MuSC pool size, with the deficit emerging during early postnatal development, whereas increasing FGF6 availability during this period expanded the pool [131]. Notably, FGF6 delivery to adult muscle did not significantly increase MuSC numbers, indicating that this pool-scaling effect is restricted to an early postnatal window [131]. These findings support a temporally restricted role for FGF6 in establishing the adult MuSC pool, with additional context-dependent effects during muscle regeneration.

A distinct niche-derived interaction has been proposed for FGF7. Single-cell analysis of porcine skeletal muscle predicted communication from fibro-adipogenic progenitors (FAPs) to MuSCs through FGF7–FGFR2 [132]. Recombinant FGF7 increased proliferation in primary porcine MuSCs and C2C12 cells, and FGFR2 knockdown attenuated this response in both myogenic systems. In primary porcine MuSCs, the proliferative response showed a nonlinear concentration dependence, with the response increasing across lower concentrations but no longer evident at the highest concentration tested [132]. Intramuscular administration of FGF7 after cardiotoxin injury also increased the proportions of Pax7-positive cells and 5-ethynyl-2′-deoxyuridine-positive/Pax7-positive cells, together with the abundance of embryonic myosin heavy chain-positive regenerating fibers in mice [132]. These findings support an FGF7-dependent proliferative effect but do not yet establish an endogenous FAP-to-MuSC FGF7–FGFR2 circuit in vivo, because cell-specific genetic validation is lacking and FGFR2 expression is low or undetectable in available studies of adult mouse MuSC datasets [13,55]. The FGF7–FGFR2 signaling axis is therefore best considered an emerging niche interaction whose contribution may vary with species and regenerative state.

### 3.3. FGF Signaling During MuSC Differentiation and Fusion

After expansion, a substantial fraction of MuSC progeny must withdraw from the cell cycle, activate the myogenic differentiation program, and fuse to form new myotubes or repair damaged myofibers [135,136]. Continued exposure to mitogenic FGFs can sustain proliferation and suppress commitment to terminal differentiation, whereas attenuation or redistribution of these inputs permits lineage progression [137,138,139,140,141]. This transition therefore reflects reconfiguration rather than complete loss of FGF signaling, involving changes in receptor competence, extracellular ligand presentation, and downstream signaling dynamics.

The differentiation-inhibitory effects of sustained FGF signaling have been demonstrated most clearly in myoblast and primary myogenic culture systems. Classic studies showed that FGF2 can prevent cells in G1 from committing to terminal differentiation [137]. Increased FGFR1 availability likewise favors proliferation and delays differentiation, whereas reduced functional receptor signaling decreases proliferation and facilitates differentiation [138,139]. Several intracellular mechanisms contribute to this effect. Src homology 2 domain-containing protein tyrosine phosphatase 2 (SHP-2) enhances FGFR-mediated repression of myogenic gene expression in C2C12 cells, whereas SPRY2 overexpression limits FGF2 signaling and permits differentiation despite continued ligand exposure [140,141]. FGF2 also suppresses the pro-myogenic long intergenic noncoding RNA activator of myogenesis (Linc-RAM) in a MyoD-dependent manner; restoration of Linc-RAM attenuates FGF2-induced differentiation defects in C2C12 and ex vivo MuSC systems [142]. Notably, downstream ERK requirements vary among myogenic models: in MM14 cells, ERK1/2 was required for FGF-dependent proliferation but was not required for FGF-mediated repression of myogenic differentiation [143].

Extracellular ligand presentation is remodeled in parallel. Perlecan, which binds and retains differentiation-inhibitory FGF2, is downregulated during myogenic differentiation [144]. Persistent syndecan-1 expression inhibits differentiation through an FGF2-dependent mechanism, whereas glypican-1 sequesters FGF2 in lipid-raft domains and reduces its access to signaling-competent FGFRs, thereby permitting differentiation [95,145]. Sulf1- and Sulf2-mediated remodeling of HS provides an in vivo example of this regulatory layer: Sulfs attenuate FGF2 signaling in activated MuSCs, and combined Sulf deficiency delays myogenic differentiation during muscle regeneration [91]. Differentiation can therefore be enabled by altered retention and presentation of existing FGF ligand as well as by changes in ligand abundance.

Other FGFs illustrate additional ligand-, dose-, and model-dependent effects. Extracellular FGF1 inhibits differentiation in rat myogenic cells, whereas intracellular FGF1 expression promotes differentiation in the same model, emphasizing the importance of signaling mode [146]. FGF9 inhibits differentiation in both C2C12 and cultured human skeletal muscle cells. In C2C12 cells, this effect is accompanied by reduced myogenin and myosin heavy chain expression and increased myostatin expression, whereas substantially higher FGF9 concentrations are required to inhibit differentiation in human cells [147]. FGF16 and FGF20 likewise inhibit C2C12 differentiation, but significant effects were observed only at the highest concentration tested [147]. In contrast, FGF21 overexpression promotes cell-cycle exit and differentiation in C2C12 cells through a proposed p53–p21 mechanism [148]. Because this evidence derives from a cell line overexpression model and does not establish a signaling-competent FGFR–β-Klotho complex in adult MuSCs, the relevance of FGF21 to direct MuSC regulation during regeneration remains unresolved [48,64]. FGF13 overexpression also inhibits C2C12 proliferation and differentiation in association with reduced SPRY1 and altered ERK1/2 signaling, but its role in adult MuSCs remains unclear [36].

In vivo receptor studies provide an important counterpoint to the differentiation-inhibitory effects observed under sustained FGF exposure in culture. Whole-body *Fgfr4* deficiency is associated with delayed and disorganized myogenic differentiation during regeneration, supporting a contribution of FGFR4 to coordinated tissue reconstruction [111]. MuSC-specific combined deletion of *Fgfr1* and *Fgfr4* also causes precocious terminal differentiation rather than simply blocking myogenesis [13]. Together, these findings indicate that FGFR signaling can preserve progenitor competence and prevent premature differentiation even though sustained mitogenic FGF2 exposure inhibits differentiation in culture. FGF6 further illustrates the state dependence of this response. Studies in regenerating adult soleus muscle using *Fgf6*-deficient mice and recombinant FGF6 rescue support a dose-dependent model in which higher FGF6 levels favor myogenic proliferation, whereas lower levels influence differentiation and muscle phenotype through calcineurin-dependent regulation [134]. In developing tongue muscle, a transforming growth factor-β (TGF-β)–SMAD4–FGF6 cascade contributes to terminal differentiation and fusion, and exogenous FGF6 partially rescues the fusion defect caused by myogenic *Smad4* loss [134,149]. These findings support dose- and context-dependent roles for FGF6 in late myogenesis rather than a uniform pro-differentiation effect. Evidence that FGF signaling directly regulates the core membrane-fusion machinery remains less developed than evidence for its effects on proliferation and differentiation timing. Changes in myotube formation after FGF manipulation can arise from altered cell-cycle exit, lineage commitment, cell number, or ECM organization. FGF signaling is therefore better viewed as shaping the competence and timing of cells entering fusion than as exerting a uniform pro-fusion or anti-fusion effect.

### 3.4. FGF Signaling During MuSC Self-Renewal and Return to Quiescence

Regeneration also requires a subset of MuSC progeny to avoid terminal differentiation and restore the quiescent MuSC pool [15,107]. Ex vivo myofiber studies show that activated Pax7-positive progeny adopt divergent fates: most proceed toward differentiation, whereas a subset retains Pax7, downregulates MyoD, and reacquires a quiescence-like state [16,150,151]. Successful self-renewal therefore requires both withdrawal from the injury-associated proliferative program and restoration of niche-dependent quiescent identity [9,16].

SPRY1 provides the clearest direct connection between attenuation of RTK signaling and the return of MuSCs to quiescence. *Spry1* is expressed in quiescent MuSCs, decreases after activation, and is re-induced in Pax7-positive cells that return to quiescence [81,151]. Conditional deletion of *Spry1* in cycling Pax7-lineage cells reduces the number of self-renewed MuSCs after injury and compromises restoration of the quiescent pool [81,151]. Temporal deletion experiments further indicate that the requirement for SPRY1 is greatest during the regenerative window in which the self-renewing pool is being re-established [81]. SPRY1-dependent attenuation therefore supports the return to quiescence, and pharmacological MEK inhibition rescues the defect in the return to quiescence of *Spry1*-deficient myogenic cells in vitro, implicating ERK as an important downstream effector [81]. Because SPRY1 is not exclusive to FGFRs, SPRY1-mediated feedback is best viewed as a broader RTK regulatory mechanism through which FGF and other growth factor responses are restrained as regeneration resolves.

Return to quiescence does not appear to require complete loss of FGFR activity. Combined MuSC-specific deletion of *Fgfr1* and *Fgfr4* reduces self-renewal and drives activated lineage cells toward premature cell-cycle exit and terminal differentiation [13]. Taken together with the SPRY1 findings, these results suggest that restoration of the MuSC pool depends on a balance between sufficient FGFR activity to preserve progenitor competence and feedback restraint that limits persistent RTK signaling.

Restoration of niche architecture also contributes to MuSC self-renewal. During regeneration, MuSCs locally remodel the basal lamina, including deposition of laminin-α1 and laminin-α5, and disruption of this remodeling impairs MuSC expansion and self-renewal [110]. Syndecan-3 deficiency likewise alters MuSC–niche interactions, homing, and progenitor homeostasis [98]. Although these studies do not directly establish FGF-specific effects during re-quiescence, they support niche reconstruction as a determinant of the signaling context in which the MuSC pool is restored.

Across the regenerative sequence, genetic studies most clearly establish a combined requirement for FGFR1 and FGFR4 in quiescence and self-renewal, an FGFR1-dependent mitogenic response during expansion, and SPRY1-mediated RTK feedback during the return to quiescence [13,55,81]. The functions of individual ligands remain more dependent on experimental model and regenerative context. Nevertheless, the predominant interpretation of FGF signaling shifts from FGFR-dependent maintenance under feedback restraint in quiescence to expansion-promoting activity after injury, followed by signaling reconfiguration during lineage progression and renewed feedback restraint as the MuSC pool is restored.

## 4. FGF Signaling in Aging and Pathological Muscle Remodeling

**Figure 3:**
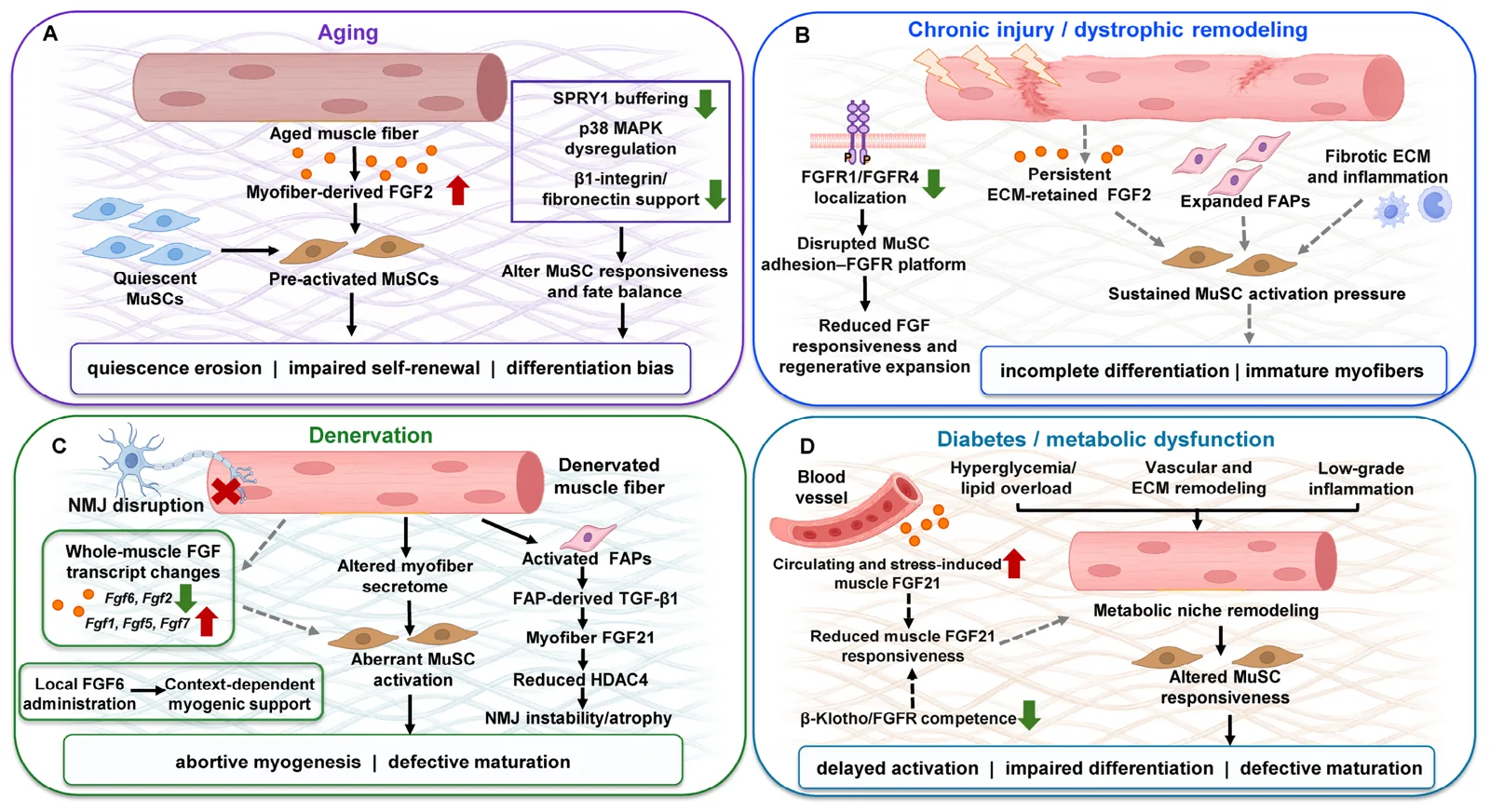
Altered FGF signaling in aging and pathological muscle remodeling. Aging and pathological muscle remodeling alter the production, presentation, reception, and interpretation of FGF signals during MuSC-mediated repair. (**A**) In aging, increased myofiber-derived FGF2 promotes MuSC pre-activation, whereas reduced SPRY1 buffering, p38 MAPK dysregulation, and diminished β1-integrin/fibronectin support alter MuSC responsiveness and fate balance. (**B**) During chronic injury and dystrophic remodeling, persistent ECM-retained FGF2, expanded FAPs, and fibrotic ECM and inflammation contribute to sustained MuSC activation pressure and impaired lineage progression. In parallel, reduced membrane localization of FGFR1/FGFR4 disrupts the MuSC adhesion–FGFR platform, reducing FGF responsiveness and regenerative expansion. (**C**) Denervation alters the myofiber secretome and whole-muscle FGF transcript profile, with secretome remodeling contributing to aberrant MuSC activation and defective myogenic maturation. A FAP–transforming growth factor β1 (TGF-β1)–myofiber FGF21–histone deacetylase 4 (HDAC4) pathway promotes neuromuscular junction (NMJ) instability and myofiber atrophy, whereas local FGF6 administration provides context-dependent myogenic support. (**D**) In diabetes and metabolic dysfunction, metabolic niche remodeling alters MuSC responsiveness, while elevated FGF21 and reduced β-Klotho/FGFR competence accompany reduced FGF21 responsiveness in mature skeletal muscle. The evidence-coding scheme is the same as that used in Figure 2.

During successful regeneration, the state-dependent effects of FGF signaling are coordinated with an ordered regenerative program and a competent local niche. This coordination is altered during aging-associated decline and in disease or stress contexts, such as chronic injury, dystrophic remodeling, denervation, and diabetes or metabolic dysfunction [15,107]. In these settings, persistent inflammation, ECM remodeling, neural input loss, vascular insufficiency, and metabolic dysregulation modify FGF ligand sources and accessibility, receptor competence, and downstream signal interpretation [114,152]. These changes reshape the contribution of FGF-related inputs to regenerative outcomes and tissue remodeling [15,94,153]. Figure 3 summarizes these context-specific changes in FGF signaling and their consequences for MuSC function and muscle remodeling.

### 4.1. Aging

Aging progressively alters both MuSC state and the niche conditions under which FGF signals are interpreted [9,12,114]. Aged MuSCs do not merely decline in number. They progressively deviate from a stable and reversible quiescent state, with alterations in cell cycle control, proteostasis, mitochondrial function, and stress responses [154,155,156]. At very advanced age, a subset of MuSCs can enter a pre-senescent or senescence-like state characterized by p16^INK4a^ derepression, DNA damage accumulation, and defective activation after injury [157,158]. These age-dependent changes collectively alter the cellular context in which MuSCs interpret FGF signals [9,15].

The best-characterized age-associated FGF input is myofiber-derived FGF2. Chakkalakal and colleagues showed that aged myofibers express FGF2 under uninjured homeostatic conditions [15]. This niche-derived FGF2 can drive a subset of MuSCs out of quiescence and reduce their self-renewal capacity [9,15]. During acute injury in young muscle, FGF2 contributes to early MuSC activation and expansion [15]. However, in uninjured aged muscle, elevated FGF2 is encountered within a niche that normally maintains quiescence [9,15]. Its effect is therefore determined not simply by ligand identity but by timing and niche context. Persistent homeostatic exposure to FGF2 is more likely to promote inappropriate quiescence exit and erosion of the stem cell pool than to support an ordered sequence of expansion, differentiation, and return to quiescence [9,114].

Whether this FGF2 pressure leads to depletion of the MuSC pool also depends on the ability of MuSCs to restrain FGF/RTK output [15]. SPRY1 is an important negative regulator of FGF/RTK signaling and limits excessive ERK activity during quiescence maintenance and return to quiescence [81]. Increased myofiber-derived FGF2 in aged muscle places greater demand on this feedback capacity [15]. Studies of aged human MuSCs suggest that age-associated epigenetic changes at the *SPRY1* locus reduce *SPRY1* expression and impair return to quiescence [159]. Functional studies further show that enhanced FGF signaling or loss of *Spry1* promotes MuSC quiescence exit and stem cell pool depletion, whereas inhibition of FGFR1 signaling or restoration of SPRY1 expression mitigates this process [15,159].

Aging also alters how MuSCs interpret FGF input. Aged MuSCs show reduced proliferative responses to FGF2 under injury or culture conditions, along with reduced self-renewal and increased differentiation bias [72]. This apparent paradox with elevated homeostatic FGF2 in aged muscle reflects the coexistence of mistimed ligand input and altered signaling competence [15,72]. Studies of p38α/β MAPK provide mechanistic insight into this altered responsiveness [124]. In young MuSCs, spatially asymmetric p38α/β MAPK activation directs divergent daughter cell fates [125]. In aged MuSCs, elevated and mislocalized p38α/β MAPK activity promotes MyoD expression in both daughter cells, biasing differentiation and reducing self-renewal [72,125]. Accordingly, moderate inhibition of p38α/β MAPK or modulation of FGFR1-associated signaling improves self-renewal defects in aged MuSCs [72].

Changes in niche structure further impair FGF responsiveness. In aged MuSCs, altered β1-integrin activity and localization reduce sensitivity to FGF2 [93]. This contributes to diminished proliferation, premature differentiation, and impaired self-renewal; activation of β1-integrin can partially restore FGF2 responsiveness and regenerative output [93]. Fibronectin, a key ECM component of the MuSC niche, is also reduced in aged muscle [94]. Loss of fibronectin compromises MuSC maintenance and regenerative capacity, whereas fibronectin supplementation improves regeneration in aged muscle [94]. Syndecan-4 and other HSPGs may further influence FGF responsiveness by regulating ligand presentation and FGFR complex assembly, although their specific contributions to mammalian MuSC aging remain less well defined [86,99,160].

Aging-associated FGF activity also extends beyond MuSC quiescence control to stromal fate and niche remodeling. FGF2-dependent induction of miR-29a suppresses the adipogenic inhibitor secreted protein acidic and rich in cysteine (SPARC) in aged mouse and human skeletal muscle, thereby promoting FAP adipogenesis and intramuscular fat formation in experimental models [161]. Different FGF family members, however, need not produce equivalent outcomes in the aged niche. As discussed in Section 3.2, FGF7–FGFR2 signaling has been proposed as an interaction between FAPs and myogenic cells [132]. In a D-galactose-induced aging model, exogenous FGF7 improved myofiber size and exercise performance [132]. Whether endogenous FAP-derived FGF7 is required during natural aging or operates similarly in human MuSCs remains unknown. FGF signaling in aged muscle therefore depends on the ligand source, timing, niche state, and MuSC responsiveness rather than being uniformly beneficial or detrimental.

### 4.2. Chronic Injury and Dystrophic Remodeling

In chronic injury and dystrophic muscle, the regenerative program is repeatedly initiated but fails to resolve in an orderly manner [162,163]. Chronic muscle injury is characterized by recurrent myofiber damage, incomplete repair, persistent inflammation, and abnormal ECM deposition [162,164]. These conditions expose MuSCs to sustained activation pressure, repeated demand for progenitor expansion, incomplete differentiation, and pathological niche feedback [9,162]. Duchenne muscular dystrophy (DMD) is a prototypical example. Its primary pathology arises from dystrophin deficiency, which disrupts sarcolemma–cytoskeleton–ECM coupling and increases susceptibility to myofiber damage, necrosis, and inflammation [165,166,167]. Persistent degeneration–regeneration cycles gradually generate a pathological niche characterized by inflammatory infiltration, FAP expansion, fibrosis, and fatty deposition [164,168]. Dystrophin is also expressed in activated MuSCs and contributes to cell polarity, spindle orientation, and asymmetric division [169]. Its loss impairs myogenic progenitor generation and regenerative myogenesis [169,170,171].

FGF2 provides an important example of altered ligand presentation in the dystrophic niche [172]. In normal skeletal muscle, FGF2 can localize to the endomysium and the adjacent heparin-rich basal lamina. In *mdx* muscle, ECM-bound FGF2 is increased relative to control muscle and is associated with sustained MuSC activation and regenerative responses [172]. Increased FGF2 localization can be detected before overt degeneration–regeneration cycles become prominent, linking altered FGF2 localization to changes associated with dystrophin deficiency at the interface between the sarcolemma and basal lamina [172]. FGF2 dysregulation in dystrophic muscle therefore involves not only ligand abundance but also altered storage, retention, and accessibility within the ECM. At the same time, genetic evidence indicates that FGF activity is not simply a pathological excess. Combined loss of FGF2 and FGF6 exacerbates dystrophic changes in *mdx* mice and reduces the migratory capacity of FGF-deficient myoblasts [163]. FGF2-related signaling thus occupies a dual position: it remains part of compensatory regenerative responses while becoming persistently available within the dystrophic ECM.

Effective conversion of FGF signaling into regenerative output during chronic injury also depends on receptor localization and membrane-associated signaling platforms. Although *Sgca*-null mice model sarcoglycanopathy rather than DMD directly, they provide a mechanistic example of how disruption of sarcoglycan-associated membrane organization alters FGF2–FGFR1 responsiveness in dystrophic muscle [173]. During myogenic progenitor proliferation, α-sarcoglycan (SGCA) forms a complex with FGFR1 and stabilizes the receptor at the plasma membrane. Loss of *Sgca* reduces membrane-localized FGFR1, blunts FGF2-induced cyclin D and ERK1/2 responses, and impairs clonal expansion, whereas SGCA re-expression rescues the low-proliferation phenotype [173]. Studies of Cdon extend this principle to membrane-proximal adhesion and receptor organization during regeneration after repeated injury [174,175]. MuSC-specific Cdon deletion impairs regeneration after repeated cardiotoxin injury, with increased immature myofibers and fibrosis, and reduced regenerated myofiber cross-sectional area [14]. Mechanistically, Cdon deficiency reduces FGF2-induced ERK1/2 activation, decreases the cell-surface localization of FGFR1 and FGFR4, and disrupts activated β1-integrin localization [14]. These findings identify FGFR localization and adhesion-platform integrity as important determinants of FGF responsiveness during chronic or repeated injury.

Changes in cellular composition and ECM state further modify how FGF inputs are presented and interpreted [162]. During acute regeneration, FAPs can support repair through transient expansion, secretion of regenerative factors, and ECM production [176,177,178]. In DMD-like chronic injury, persistent inflammation and TGF-β-associated fibrotic signaling instead favor fibrotic or adipogenic trajectories in FAPs and other stromal cells [164,170,179,180]. ECM accumulation and tissue stiffening can impair maturation of newly formed myofibers while altering the accessibility and diffusion range of locally retained FGF ligands [172,181,182]. FGF signals in this setting are therefore received within a niche characterized by persistent inflammation, stromal activation, ECM remodeling, and altered membrane organization rather than within the transient environment of acute repair. FGF-related consequences can also extend beyond the local niche. Muscle-derived FGF21 is elevated in dystrophin/utrophin double-knockout mice and has been linked to pathological muscle–bone communication [183,184]. This systemic stress response extends the consequences of dystrophic FGF signaling beyond local MuSC regulation.

### 4.3. Denervation

Denervation alters FGF biology through a mechanism distinct from acute myofiber injury because it primarily disrupts motor neural input, neurotrophic support, and the structural stability of the neuromuscular junction (NMJ) [114,185,186,187]. Loss of innervation reduces myofiber contractile activity and alters metabolic state. It is also accompanied by ECM remodeling, interstitial cell activation, inflammatory responses, and progressive NMJ disassembly [188,189,190]. Denervated muscle therefore represents an abnormal signaling niche shaped by neural input loss, altered myofiber state, stromal responses, and structural degeneration rather than a conventional regenerative niche after acute injury.

Denervation alters MuSC state and responsiveness to regenerative stimuli, but this does not eliminate intrinsic myogenic potential [191,192]. Earlier ex vivo myofiber studies showed that long-term denervation alters the expression of proliferation- and differentiation-associated markers in MuSCs and reduces their ability to re-enter proliferative and differentiation programs in culture [191]. A more direct functional study showed that even after prolonged denervation, MuSCs retain the capacity for activation, myogenic differentiation, self-renewal, and regeneration [192]. Transplantation experiments further showed that MuSCs isolated from denervated muscle can form new myofibers and reoccupy their anatomical niche when transferred to a normal physiological context [192]. Within denervated muscle, however, activated MuSCs frequently fail to complete effective terminal maturation, resulting in abortive myogenesis and defective maturation of regenerated myofibers [193,194]. These findings suggest that denervation primarily impairs the tissue niche that converts MuSC activity into effective repair. A recent study of the myofiber secretome supports this niche-driven interpretation [195]. Denervation markedly alters the myofiber transcriptome and secretome and reshapes the MuSC niche. These changes lead to premature or abnormal MuSC activation and to increased proportions of Pax7-positive, MyoD-positive, and myogenin-positive cells [195]. When muscle injury occurs in the context of denervation, regenerated myofiber maturation is delayed and tissue repair quality is reduced [195]. Increased myofiber-derived TGF-β1, osteopontin, and other secreted factors are associated with these abnormalities, suggesting that denervation-induced MuSC dysfunction reflects coordinated remodeling of the secretory environment rather than the action of a single factor [195]. Denervation-activated FAPs also contribute to pathological remodeling through STAT3–interleukin-6 (IL-6) signaling that promotes myofiber atrophy and fibrosis [195,196].

Within this altered niche, FGF signaling is best viewed as one component of state-dependent remodeling rather than as an isolated MuSC fate determinant. Whole-muscle mRNA analysis of rat plantaris muscle showed that denervation decreases *Fgf2* and *Fgf6* mRNA levels, while increasing *Fgf1*, *Fgf5*, and *Fgf7* mRNA levels [187]. When denervated muscle is subjected to compensatory overload, *Fgf2*, *Fgf7*, and related growth factor transcripts increase relative to denervation alone [187]. These whole-muscle data demonstrate reorganization of the local FGF transcript landscape without identifying individual cellular sources or direct MuSC effects. FGF6 provides more direct functional evidence that FGF output depends on ligand dose and receptor context [126,197]. Dose–response experiments in C2C12 cells and primary myoblasts showed that higher FGF6 concentrations preferentially engaged FGFR1-associated signaling and supported viability and migration, whereas lower concentrations favored FGFR4–ERK1/2 signaling and myogenic differentiation [197]. In a sciatic nerve injury model, local FGF6 administration increased myofiber cross-sectional area and restored FGFR4- and ERK-associated myogenic signaling in denervated muscle [197]. These data suggest that FGF6 can support myogenic repair under selected denervation-associated conditions, with its output shaped by concentration, receptor context, and local tissue state.

By contrast, FGF21 provides a clear example of maladaptive FGF family signaling after denervation. FGF21 is strongly induced in denervated skeletal muscle and promotes denervation-associated muscle wasting primarily through local autocrine or paracrine mechanisms [198]. Muscle *Fgf21* knockdown or deletion attenuates atrophy and preserves NMJ innervation. Conversely, muscle-specific FGF21 overexpression reduces NMJ innervation and exacerbates atrophy [198]. Mechanistically, denervation-activated FAPs release TGF-β1, which induces FGF21 expression in mature myotubes through the c-Jun N-terminal kinase (JNK)–c-Jun axis. FGF21 then reduces cytoplasmic histone deacetylase 4 (HDAC4), compromises NMJ maintenance, and promotes myofiber atrophy [198]. This FAP–TGF-β1–FGF21–HDAC4 axis acts primarily through regulation of mature myofibers and the NMJ rather than through direct control of MuSC fate, illustrating how FGF family signaling can be redirected toward a pro-atrophic output in the denervated niche.

### 4.4. Diabetes and Metabolic Dysfunction

Diabetes and related metabolic dysfunction reshape both the local regenerative niche and the systemic metabolic environment [199,200,201]. Skeletal muscle is a major site of insulin-stimulated glucose disposal and a key metabolic tissue affected by diabetes [202]. Hyperglycemia, insulin resistance, obesity-associated lipid overload, chronic low-grade inflammation, vascular dysfunction, and ECM remodeling collectively affect myogenic and non-myogenic cell populations during repair [200,203,204,205]. These systemic and local alterations create a metabolically altered regenerative niche in which FGF inputs may be interpreted differently from those encountered during acute injury repair.

At the level of MuSC-mediated regeneration, diabetes and obesity can impair MuSC activation, proliferation, or differentiation, as well as the subsequent growth and maturation of regenerated myofibers [199,206]. However, the phenotype varies across experimental models, disease stages, and injury paradigms. In some settings, regenerative impairment is not explained by MuSC number alone; functional defects in MuSCs and subsequent regenerative progression may also contribute [207,208]. Diabetes-associated MuSC dysfunction can also involve durable cell-autonomous alterations [209,210]. Consistent with these findings, primary myogenic cells derived from individuals with type 2 diabetes can retain abnormal inflammatory cytokine responses and impaired myogenic differentiation ex vivo, while studies of human skeletal muscle have identified diabetes-associated epigenetic and microRNA changes [211,212,213,214]. At the niche level, diabetes disrupts inflammatory resolution, FAP behavior, vascular support, ECM turnover, and collagen remodeling, while increasing oxidative stress and a fibrotic tendency [215,216]. These extracellular and metabolic changes reshape the context in which FGF ligand presentation, receptor competence, and downstream signaling are interpreted.

Among FGF family members, FGF21 is most closely linked to metabolic dysfunction [48,217,218]. Unlike heparin-binding paracrine FGFs, FGF21 acts as an endocrine and stress-responsive regulator of glucose, lipid, and energy metabolism [219]. Although the liver is generally considered the major source of circulating FGF21, skeletal muscle can also induce FGF21 expression in selected contexts, including acute insulin stimulation, mitochondrial dysfunction, and impaired mitochondrial fatty acid oxidation [220,221,222]. Muscle-derived FGF21 is therefore better viewed in this setting as a stress- or signal-responsive metabolic output than as a conventional local MuSC expansion factor [218,223]. In insulin-resistant human skeletal muscle cells and diet-induced obesity models, elevated FGF21 can coexist with reduced β-Klotho expression and decreased phosphorylation of FGFR, FRS2α, and ERK1/2 [218,224]. This dissociation between ligand induction and signaling competence illustrates how metabolic state can impair FGF21 responsiveness.

Paracrine FGFs provide a complementary example of metabolic regulation via FGF signaling in mature skeletal muscle [225]. In experimental models, FGF1 and FGF4 can act on mature skeletal muscle expressing abundant FGFR1c but little or no β-Klotho, promoting glucose disposal through β-Klotho-independent mechanisms [225]. In diabetic mouse models, these effects have been linked to insulin-independent AMP-activated protein kinase (AMPK) activation and increased glucose transporter 4 (GLUT4) availability at the myofiber surface [225]. These findings establish metabolic effects of FGF signaling in mature skeletal muscle rather than direct restoration of MuSC fate transitions. Their relevance to regeneration is more likely to be indirect, through altered glucose handling and changes in the metabolic niche in which MuSC-mediated repair occurs [199].

## 5. Malignant Rewiring of Myogenic FGF Signaling in Rhabdomyosarcoma

RMS provides a distinct malignant context in which to extend the state-dependent FGF framework from MuSC-mediated regeneration to oncogenic disruption of myogenic cell-state control. RMS exhibits skeletal muscle lineage programs and comprises heterogeneous malignant cell states with progenitor-like, proliferative, and differentiated features [226]. These features make RMS informative for understanding how signaling networks that normally coordinate myogenic progenitor expansion and lineage progression can be redirected toward persistent proliferation and incomplete differentiation. Unlike regenerative myogenesis, in which FGF output is reconfigured as progenitors exit expansion, oncogenic programs can incorporate FGF signaling into mechanisms that stabilize malignant myogenic states.

FGFR4 provides a well-characterized example of this rewiring. Activating mutations within the FGFR4 kinase domain occur in a subset of primary RMS tumors and enhance receptor autophosphorylation and downstream STAT3 signaling while promoting tumor growth and metastatic behavior in experimental models [227]. FGFR4 is also a direct transcriptional target of the PAX3-FOXO1 fusion oncogene in fusion-positive RMS (FP-RMS), although increased expression of wild-type FGFR4 alone does not reproduce the full proliferative, transforming, and differentiation-blocking effects of PAX3-FOXO1 in primary myoblasts [228]. More recently, Kalita et al. used engineered human muscle progenitors and patient-derived preclinical models to show that PAX3-FOXO1 and PAX7-FOXO1 reorganize chromatin architecture and establish regulatory interactions at oncogenic loci, including FGFR4 [229]. FGFR4 inhibition reduced RMS cell proliferation and clonogenic growth and showed antitumor activity in selected fusion-positive models [229]. These findings place FGFR4 within a broader malignant cell-state program in which receptor output can be reinforced by oncogenic transcriptional regulation or directly activated by receptor mutation.

Ligand-dependent signaling provides a complementary form of malignant FGF rewiring. FGF7 and FGFR2 are expressed at higher levels in FP-RMS than in fusion-negative RMS, and PAX3-FOXO1-positive cells maintain FGF7 secretion and MAPK activity during growth factor deprivation [230]. Genetic suppression of either FGF7 or FGFR2 reduces viability of tumor cells, whereas pan-FGFR inhibition suppresses FP-RMS xenograft growth. In vitro, FGFR inhibition also enhances sensitivity to SN-38, the active metabolite of irinotecan [230]. Together, these findings support a functional FGF7–FGFR2 autocrine circuit and broader FGFR dependence in FP-RMS. This circuit provides an informative comparison with the candidate FAP-derived FGF7–FGFR2 interaction discussed in Section 3.2. In the regenerative niche, FAP-derived FGF7 has been proposed as a stromal input acting on myogenic cells; in FP-RMS, the same ligand–receptor pair can instead operate as a tumor-associated autocrine circuit. FGF8 further illustrates how ligand–receptor coupling can change with malignant state. As a ligand regulated by PAX3-FOXO1, FGF8 contributes to tumorigenicity in FP-RMS models and is associated with FGFR-dependent MAPK signaling [231,232]. Persistent FGF8 expression in an experimental PAX3-FOXO1-independent recurrent state was accompanied by a shift toward FGFR1-dependent signaling [231]. These findings illustrate how ligand availability, receptor usage, and the state of the responding myogenic cell jointly determine the biological output of FGF signaling.

FGF signaling can become more autonomous through structural alteration of the receptor. FGFR1-ANK1 and FGFR1-TACC1 fusions have been identified in molecularly characterized RMS cases, with both predicted fusion proteins retaining the FGFR1 tyrosine kinase domain, consistent with oncogenic kinase activation [233]. Although rare, these rearrangements illustrate how malignant transformation can partly bypass the normal dependence of FGF signaling on regulated ligand presentation and receptor activation. RMS therefore represents oncogenic rewiring rather than a simple increase in physiological FGF signaling. Across these mechanisms, changes in receptor activation, ligand–receptor organization, and malignant cell state can generate persistent FGF dependencies that support tumor growth and survival within incompletely differentiated myogenic states. These dependencies provide a rationale for molecularly stratified evaluation of FGFR-targeted therapeutic strategies. Representative FGF signaling relationships across MuSC fate transitions and the aging-associated, pathological, and malignant contexts discussed above are summarized in Table 2.

## 6. Perspectives

The state-dependent nature of FGF signaling makes stage-appropriate modulation the central objective of FGF-directed regenerative strategies. The genetic and functional evidence reviewed above argues against uniform FGFR activation or suppression throughout regeneration. Excessive or mistimed FGF2–FGFR1 activity can destabilize MuSC quiescence in aged muscle, whereas broad receptor inhibition could compromise signaling required for progenitor competence and regenerative progression [13,15,55]. Effective FGFR modulation therefore depends on the regenerative stage, the identity of the target cell, and the reversibility of the intervention. Local or cell-targeted delivery, receptor-selective agents, tunable exposure schedules, and modulation of ligand presentation or intracellular feedback may provide greater control than systemic pan-FGFR manipulation. In RMS, by contrast, FGFR inhibition represents a distinct application in oncology, for which molecular evidence of receptor alteration or pathway dependence should guide therapeutic selection [227,228,229,230,231,232,233].

Temporal control is equally important for regenerative applications. Controlled FGF2 delivery combined with myoblast transplantation improved donor-cell survival and promoted muscle regeneration in a rat muscle-defect model, demonstrating that local growth-factor exposure can be manipulated to support repair [234]. Greater or more sustained FGF activity, however, does not necessarily improve all dimensions of regeneration. In a murine volumetric muscle loss model, FGF2-releasing aligned nanofibrillar collagen scaffolds produced endpoint-specific effects: twitch force was higher with FGF2 delivery alone than with combined FGF2 delivery and exercise, myofiber density was not significantly improved, and increased reinnervation was associated with scaffold implantation plus exercise rather than FGF2 delivery [235]. Similarly, transplantation of FGF2-overexpressing myoblasts after rat muscle injury increased proliferation and reduced apoptosis but did not significantly improve muscle force and was accompanied by increased adipogenic responses [236]. These findings argue against equating sustained ligand availability with successful regeneration. For interventions intended to support early MuSC activation or progenitor expansion, a plausible design objective is a defined early exposure window followed by sufficient attenuation to permit differentiation, fusion, and restoration of the MuSC pool. Biomaterials capable of tuning ligand retention and release, including affinity-based depots and degradable hydrogels, provide practical platforms for testing this principle [237,238]. Relevant evaluation parameters include release kinetics, the local concentration of bioavailable ligand, receptor engagement, and the duration and termination of downstream signaling, rather than only the nominal amount of FGF delivered.

Safety considerations must likewise reflect the state- and tissue-dependent functions of the FGF network. Prolonged pathway activation may sustain proliferative signaling, delay differentiation, erode the quiescent MuSC reserve, or redirect stromal and vascular remodeling, whereas broad systemic inhibition may interfere with physiological FGFR functions outside the regenerative compartment. The oncogenic FGFR dependencies identified in RMS further underscore the importance of spatially and temporally controlled pathway modulation. Clinical experience with systemic FGFR inhibitors in oncology provides an additional safety context: on-target hyperphosphatemia and mucosal, cutaneous, and ocular toxicities are well documented [239,240]. Although regenerative interventions would differ substantially in route, dose, target tissue, and treatment duration, these observations favor local and transient exposure whenever feasible. Preclinical safety assessment should therefore extend beyond short-term histology to encompass durable force recovery, preservation of the MuSC pool and its response to repeated injury, fibrosis and ectopic adipogenesis, vascular and neural integration, phosphate and metabolic homeostasis, and evidence of aberrant tissue growth.

Translation is also constrained by the experimental models used to define therapeutic windows. Cardiotoxin, notexin, barium chloride, and freeze injury differ in the extent of MuSC preservation, damage to the basal lamina and vasculature, inflammation, and subsequent regeneration [241]. These acute models generate synchronized and largely self-resolving injuries that capture only part of the prolonged and heterogeneous remodeling encountered in aging and chronic human muscle disease. Disease models present different limitations. The conventional *mdx* mouse retains comparatively strong regenerative capacity and develops a milder skeletal muscle phenotype than human Duchenne muscular dystrophy, whereas telomere-shortened *mdx/mTR* mice exhibit greater MuSC dysfunction and more progressive disease [242]. No single model reproduces the full combination of ligand exposure, niche remodeling, cellular competence, and temporal dynamics relevant to human FGF intervention. Effects reproduced across acute, aged, chronic, or repeated-injury settings and supported by long-term functional endpoints consequently carry greater translational weight. Primary human MuSCs and human muscle xenografts, together with human skeletal muscle organoids, provide complementary platforms in which human-specific receptor usage, dose responses, and cell-specific FGF effects can be examined [10,243]. These systems are most informative when integrated with, rather than substituted for, in vivo functional studies.

## 7. Conclusions

FGF signaling acquires different biological meanings across skeletal muscle homeostasis, acute repair, chronic remodeling, and malignant myogenic states. Its output depends on signal magnitude and duration, extracellular presentation, receptor and coreceptor competence, and the state of the responding cell. This framework helps explain why FGF signaling can contribute to quiescence maintenance or progenitor competence in one setting, promote expansion in another, and impede lineage progression or contribute to pathological remodeling when sustained or misdirected. FGF signaling is therefore best viewed as a tunable component of the regenerative niche rather than as an autonomous instruction for a single MuSC fate decision.

Translating this framework into therapeutic strategies requires not only identifying a favorable ligand or receptor but also defining the molecular target, cellular or tissue compartment, exposure window, pharmacodynamic measures of receptor engagement and signal termination, and endpoints that demonstrate durable repair without depletion of the MuSC pool. State-resolved single-cell and spatial analyses can help identify responsive cell populations and candidate therapeutic windows, but causal perturbation in disease-relevant models remains necessary to establish which relationships are therapeutically actionable. The central translational challenge is not how to maximize or minimize FGF signaling, but how to achieve the appropriate degree of pathway activity in the relevant cell for the required interval and then permit its timely termination.

## Figures and Tables

**Table 1:** Mammalian FGF subfamilies, representative ligand–FGFR pairs, and principal cofactor/coreceptor requirements.

FGF Subfamily	FGF Members	Representative Ligand–FGFR Pairs	Signaling Mode and Cofactor/Coreceptor
FGF1	FGF1, FGF2	FGF1: FGFR1b/1c, FGFR2b/2c, FGFR3b/3c, FGFR4; FGF2: FGFR1b/1c, FGFR2c, FGFR3c, FGFR4	Paracrine; HS/HSPGs
FGF4	FGF4, FGF5, FGF6	FGF4: FGFR1c, FGFR2c, FGFR3c, FGFR4 FGF5: FGFR1c, FGFR2c, FGFR3c FGF6: FGFR1c, FGFR2c, FGFR4	
FGF7	FGF3, FGF7, FGF10, FGF22	FGF3/FGF10/FGF22: FGFR1b, FGFR2b FGF7: FGFR2b	
FGF8	FGF8, FGF17, FGF18	FGF8/FGF17: FGFR1c, FGFR2c, FGFR3b/3c, FGFR4 FGF18: FGFR2c, FGFR3b/3c, FGFR4	
FGF9	FGF9, FGF16, FGF20	FGF9/FGF20: FGFR1c, FGFR2c, FGFR3b/3c, FGFR4 FGF16: FGFR2c, FGFR3c, FGFR4	
FGF15/19	FGF15/19, FGF21, FGF23	FGF15/19: FGFR1c, FGFR2c, FGFR3c, FGFR4 FGF21: FGFR1c, FGFR2c, FGFR3c FGF23: FGFR1c, FGFR3c, FGFR4	Endocrine; β-Klotho for FGF15/19 and FGF21; α-Klotho for FGF23; HS/HSPGs contribute to receptor-complex assembly
FGF11	FGF11–FGF14	No canonical ligand–FGFR pairing	Primarily intracellular

Paracrine fibroblast growth factors (FGFs) signal mainly through heparan sulfate (HS)/heparan sulfate proteoglycan (HSPG)-assisted fibroblast growth factor receptor (FGFR) activation, whereas endocrine FGFs require Klotho-containing FGFR complexes, with HS also contributing to receptor assembly and activation. FGF15 is the mouse ortholog of human FGF19. FGF11–FGF14, also known as FGF homologous factors (FHFs), are primarily intracellular proteins.

**Table 2:** Representative FGF signaling axes and regulatory mechanisms across MuSC fate transitions, aging-associated decline, pathological muscle remodeling, and rhabdomyosarcoma.

FGF Axis or Regulatory Mechanism	Ligand Source/Input	Receptor/Coreceptor/Regulator	Target Cell	Biological Context	Experimental Model	Principal Outcome	Evidence Level
FGFR1/FGFR4-dependent signaling	Endogenous local FGF inputs; ligand identity unresolved	FGFR1, FGFR4	Adult MuSCs	Quiescence/MuSC state maintenance	Inducible MuSC-specific *Fgfr1/Fgfr4* deletion in mice	Combined receptor loss causes spontaneous quiescence exit, precocious differentiation, and progressive MuSC pool depletion [13]	I
FGF2–FGFR1 signaling	Local FGF2, exogenous FGF2 in mechanistic studies	FGFR1; HS/HSPGs, β1-integrin, Cdon, fibronectin	Adult MuSCs/myogenic progeny	Injury-induced activation/proliferative expansion	Isolated myofibers, primary MuSCs, myogenic-lineage *Fgfr1* deletion, and MuSC-specific *Fgfr1/Fgfr4* deletion models	FGF2 promotes MuSC activation and proliferative expansion; FGFR1 loss markedly reduces FGF2 responsiveness, while adhesion competence further modifies the response [13,14,55,93,94,122,123,130]	I
FGF6-dependent MuSC pool scaling	Endogenous FGF6; exogenous FGF6 in temporal intervention studies	Receptor mediating pool scaling unresolved	Postnatal MuSCs	Early postnatal establishment of the adult MuSC pool	*Fgf6*-deficient mice and temporally restricted FGF6 administration	FGF6 regulates adult MuSC pool size during a restricted early postnatal window, whereas administration in adulthood does not reproduce the pool-expanding effect [131]	II
FGF7–FGFR2 signaling	FAP-derived FGF7 (single-cell prediction); recombinant FGF7	FGFR2	Primary porcine MuSCs/other myogenic cells	Proliferative expansion/regenerative niche signaling	Porcine single-cell analysis, primary porcine MuSCs, C2C12 cells, and CTX-injured mice	FGF7 promotes myogenic-cell proliferation and increases proliferating Pax7-positive cells and regenerating fibers after injury [132]	III
FGF2–HS/Sulf1/Sulf2 regulation	HS/HSPG-retained FGF2	FGFRs; HS, Sulf1, Sulf2	Activated/differentiating MuSCs and myogenic cells	Differentiation/lineage progression	Sulf1/Sulf2-deficient mice and myogenic cell models	Sulf-mediated HS remodeling attenuates FGF2 signaling in activated MuSCs and supports the proliferation-to-differentiation transition; combined Sulf deficiency delays myogenic differentiation during regeneration [91,95,144,145]	II
SPRY1-mediated FGF/RTK feedback	Local FGF and other RTK inputs	SPRY1–ERK/MAPK feedback restraint	Pax7-lineage MuSCs	Self-renewal/return to quiescence	Conditional *Spry1* deletion in cycling Pax7-lineage cells and MEK inhibition	SPRY1-mediated signal attenuation supports self-renewal and restoration of the quiescent MuSC pool after regeneration [81,151]	I
Aged-niche FGF2 signaling	Aged myofiber-derived FGF2	FGFR signaling; SPRY1, β1-integrin	Aged MuSCs	Aging-associated regenerative decline	Aged mouse muscle/MuSC models and human MuSC studies	Persistent FGF2 promotes inappropriate quiescence exit, while altered feedback and adhesion competence modify FGF responsiveness and self-renewal [15,72,93,159]	II
FGF2 availability and receptor organization	ECM-retained FGF2; exogenous FGF2 in mechanistic studies	FGFR1 (SGCA); FGFR1/FGFR4 and β1-integrin (Cdon)	Myogenic progenitors/MuSCs	Chronic/repeated injury and dystrophic remodeling	*mdx* muscle, *Sgca*-null mice, repeated-injury models, and myogenic assays	Increased ECM-retained FGF2 is associated with sustained regenerative activation, whereas disrupted receptor/adhesion organization reduces FGF responsiveness and regenerative expansion [14,172,173]	II
FGF6–FGFR1/FGFR4 signaling	Exogenous FGF6; local administration in vivo	FGFR1-associated signaling; FGFR4–ERK1/2	Myoblasts/myogenic cells	Denervation-associated remodeling	C2C12 cells, primary myoblasts, and rat sciatic nerve injury	FGF6 produces concentration-dependent receptor responses, while local administration increases myofiber size and restores FGFR4/ERK-associated signaling after nerve injury [197]	II
FAP–TGF-β1–FGF21–HDAC4 signaling	FGF21 induced in denervated myofibers downstream of FAP-derived TGF-β1	FGFR/coreceptor unresolved; HDAC4 downstream	Mature myofibers	Denervation-associated atrophy/NMJ remodeling	Muscle-specific FGF21 gain- and loss-of-function in denervated mice and myotube models	FGF21 promotes NMJ destabilization and muscle atrophy through an HDAC4-associated mechanism [198]	II
FGF21–FGFR/β-Klotho signaling competence	Circulating FGF21 and stress-induced muscle FGF21	FGFR–β-Klotho	Mature skeletal muscle/myotubes	Diabetes/metabolic dysfunction	Insulin-resistant human skeletal muscle cells and diet-induced obesity models	Elevated FGF21 coexists with reduced β-Klotho expression and attenuated FGFR–FRS2α–ERK signaling, indicating impaired FGF21 responsiveness [218,220,221,222,223,224]	III
Oncogenic FGFR4 signaling	Activating FGFR4 mutations; PAX3-FOXO1/PAX7-FOXO1-associated FGFR4 regulation	FGFR4	RMS cells/myogenic progenitors	RMS/malignant myogenic state	Primary human RMS tumors, engineered human muscle progenitors, and patient-derived preclinical models	FGFR4 activation promotes tumor growth and metastatic behavior, whereas FGFR4 inhibition suppresses proliferation and tumor growth in responsive models [227,228,229]	II
FGF7–FGFR2 autocrine signaling	Tumor-cell-derived FGF7	FGFR2–MAPK	FP-RMS cells	FP-RMS/malignant myogenic state	Human RMS cell models and xenografts	Autocrine FGF7–FGFR2 signaling supports MAPK activity and tumor-cell viability, while FGFR inhibition suppresses xenograft growth [230]	II

Evidence levels reflect the mechanistic directness of the evidence supporting each listed relationship. I, direct causal evidence from cell- or lineage-specific genetic manipulation in vivo; II, causal or functional evidence from in vivo genetic or intervention studies at the muscle, tissue, or tumor level, with complementary mechanistic support where available; III, supportive evidence without direct cell-specific in vivo validation, including primary-cell, ex vivo, human-cell, single-cell/omics, associative, or indirect intervention evidence. CTX, cardiotoxin; RTK, receptor tyrosine kinase; NMJ, neuromuscular junction; RMS, rhabdomyosarcoma; FP-RMS, fusion-positive rhabdomyosarcoma.

## Data Availability

No new data were created or analyzed in this study. Data sharing is not applicable to this article.

## Abbreviations

The following abbreviations are used in this manuscript: AKT, protein kinase B; AMPK, AMP-activated protein kinase; CBL, casitas B-lineage lymphoma; DAG, diacylglycerol; DMD, Duchenne muscular dystrophy; ECM, extracellular matrix; ERK, extracellular signal-regulated kinase; FAP, fibro-adipogenic progenitor; FGF, fibroblast growth factor; FGFR, fibroblast growth factor receptor; FHF, FGF homologous factor; FP-RMS, fusion-positive rhabdomyosarcoma; FRS2α, FGFR substrate 2α; GAB1, GRB2-associated binding protein 1; GLUT4, glucose transporter type 4; GRB2, growth factor receptor-bound protein 2; HDAC4, histone deacetylase 4; HS, heparan sulfate; HSPG, heparan sulfate proteoglycan; IL-6, interleukin 6; IL17RD, interleukin 17 receptor D; IP_3_, inositol 1,4,5-trisphosphate; JNK, c-Jun N-terminal kinase; Linc-RAM, long intergenic noncoding RNA activator of myogenesis; MAPK, mitogen-activated protein kinase; MuSC, muscle stem cell; MyoD, myoblast determination protein 1; NFAT, nuclear factor of activated T cells; NMJ, neuromuscular junction; PI3K, phosphoinositide 3-kinase; PKC, protein kinase C; PLCγ, phospholipase C gamma; RMS, rhabdomyosarcoma; RTK, receptor tyrosine kinase; SEF, similar expression to FGF; SGCA, α-sarcoglycan; SHP-2, Src homology 2 domain-containing protein tyrosine phosphatase 2; SOS, son of sevenless; SPARC, secreted protein acidic and rich in cysteine; STAT, signal transducer and activator of transcription; TGF-β, transforming growth factor beta; TRPC, transient receptor potential canonical.
